# Methodological needs in the quality and safety characterisation of nanotechnology-based health products: Priorities for method development and standardisation

**DOI:** 10.1016/j.jconrel.2021.06.016

**Published:** 2021-08-10

**Authors:** B. Halamoda-Kenzaoui, R.J. Vandebriel, A. Howarth, M. Siccardi, C.A.W. David, N.J. Liptrott, M. Santin, S.E. Borgos, S. Bremer-Hoffmann, F. Caputo

**Affiliations:** a European Commission, Joint Research Centre (JRC), Ispra, Italy; b National Institute of Public Health and the Environment (RIVM), Bilthoven, the Netherlands; c Department of Pharmacology and Therapeutics, Institute of Systems, Molecular and Integrative Biology, University of Liverpool, Liverpool, UK; d Centre for Regenerative Medicine and Devices, School of Pharmacy and Biomolecular Sciences, University of Brighton, Brighton, UK; e Department of Biotechnology and Nanomedicine, SINTEF Industry, Trondheim, Norway

**Keywords:** Nanomedicine, Nanotechnology-based products, Regulatory needs, Methodological gaps, Method standardisation, Method development, Physicochemical characterisation, Immunotoxicity assessment, ADME, Absorption, Distribution, Metabolism, Excretion, API, Active Pharmaceutical Ingredient, CARPA, Complement Activation related Pseudo-allergy, CEN, European Committee for Standardisation, EMA, European Medicines Agency, EUNCL, European Nanomedicine Characterisation Laboratory, FDA, Food and Drug Administration, ICH, International Council for Harmonisation of Technical Requirements for Pharmaceuticals for Human Use, ISO, International Standardisation Organisation, NCI-NCL, Nanotechnology Characterisation Laboratory, NPs, Nanoparticles, PBPK, Physiologically based pharmacokinetic, PCC, Physicochemical characterisation, Ph. Eur., European Pharmacopoeia, PK, Pharmacokinetic

## Abstract

Nanotechnology-based health products are providing innovative solutions in health technologies and the pharmaceutical field, responding to unmet clinical needs. However, suitable standardised methods need to be available for quality and safety assessments of these innovative products prior to their translation into the clinic and for monitoring their performance when manufacturing processes are changed. The question arises which technological solutions are currently available within the scientific community to support the requested characterisation of nanotechnology-based products, and which methodological developments should be prioritized to support product developers in their regulatory assessment. To this end, the work presented here explored the state-of-the-art methods to identify methodological gaps associated with the preclinical characterisation of nanotechnology-based medicinal products and medical devices. The regulatory information needs, as expressed by regulatory authorities, were extracted from the guidance documents released so far for nanotechnology-based health products and mapped against available methods, thus allowing an analysis of methodological gaps and needs.

In the first step, only standardised methods were considered, leading to the identification of methodological needs in five areas of characterisation, including: (i) surface properties, (ii) drug loading and release, (iii) kinetic properties in complex biological media, (iv) ADME (absorption, distribution, metabolism and excretion) parameters and (v) interaction with blood and the immune system. In the second step, a detailed gap analysis included analytical approaches in earlier stages of development, and standardised test methods from outside of the nanotechnology field that could address the identified areas of gaps. Based on this analysis, three categories of methodological needs were identified, including (i) method optimisation/adaptation to nanotechnological platforms, (ii) method validation/standardisation and (iii) method development for those areas where no technological solutions currently exist. The results of the analysis presented in this work should raise awareness within the scientific community on existing and emerging methodological needs, setting priorities for the development and standardisation of relevant analytical and toxicological methods allowing the development of a robust testing strategy for nanotechnology-based health products.

## Highlights

• Mapping of Information requirements for nanotechnology-based health products against existing methods.
• Identification of five broad areas of methodological gaps.
• Identification of promising methods that require further optimisation and standardisation.
• Highlighting areas, where method development is required.

## Graphical abstract

**Figure:**
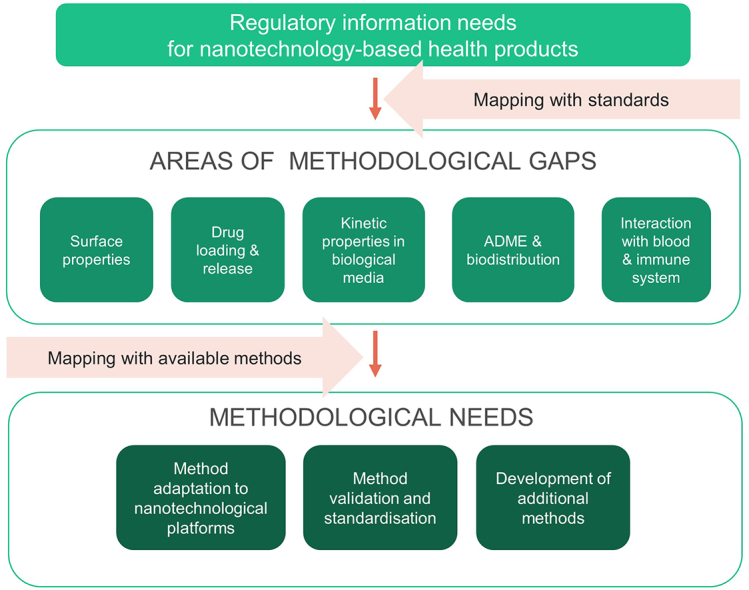

## 1 Background

Nanotechnology-based health products are an emerging class of innovative medical products and devices offering innovative therapeutic and diagnostic opportunities. Currently, in the field of nanomedicine more than 50 formulations have been approved for clinical use, including indications for cancer treatment, iron-replacement therapies, imaging agents, anaesthetics, fungal treatments, and treatments for macular degeneration [1,2]. The relevance of nanotechnology in health products was remarkably demonstrated in the field of vaccine development when the first covid-19 vaccines received market authorisation in less than 1 year. Thanks to their specific properties, nanotechnology-based systems can improve pharmacokinetic properties of classic drugs and biological products (*e.g.* nucleic acids) enhancing their therapeutic efficacy and reducing side effects. They can also offer new therapeutic possibilities, *e.g.* using physical factors such as magnetic field or radiation in oncological therapies [3]. The most common nanomedicinal formulations under investigation are liposomes and protein-bound drugs, but many studies are focusing on other innovative concepts such as lipid-based nanoparticles (NPs) for nucleic acid delivery, and metal and metal oxide NPs as radio-enhancers or magnetic resonance imaging (MRI) contrast agents. Other nanotechnological platforms include polymeric NPs, virus-like particles, micelles, extracellular vesicles, gold NPs and nanocrystals [1]. In the area of medical devices the use of nanotechnology provides improved properties for implants, dental materials, scaffolds for tissue regeneration purposes and smart diagnostic agents [4] using a broad range of nanomaterials including hydroxyapatite, iron oxide NPs, silver NPs, nanoceramics, nanocellulose and others.

Nanotechnology-based products are regulated under the legislative-regulatory frameworks of medicinal products or medical devices requiring the provision of data for quality, safety and efficacy as any other product class when applying for clinical application. However, due to their complex nature and the propensity to interact with biological systems, additional information is needed in order to assess the quality and safety of these innovative products [5]. Regulatory authorities such as European Medicines Agency (EMA) and Food and Drug Administration (FDA) have released a number of guidance documents highlighting parameters of relevance for this type of products. The described information needs are not the regulatory requirements in strict sense, but rather parameters that are considered important for quality and safety assessments of nanotechnology-based products, in addition to applicable ICH guidelines for medicinal products and CEN/ISO standards for medical devices. In order to satisfy such regulatory information needs, reliable, fit-for-purpose methods must be available and accepted by regulatory authorities. The availability of standardised and regulatory accepted methodologies would reduce the uncertainty for product developers and provide high quality data for quality and safety assessment, facilitating the regulatory process and thus clinical translation to benefit patients.

As part of the H2020 project REFINE, (www.refine-nanomed.eu), which aims to advance the regulatory science for nanotechnology-based health products, we have systematically analysed to what extent the currently available methods are sufficient to address the regulatory information needs and elucidated methodological gaps. In addition, we have identified promising methods that are currently used by product developers but are not yet taken up in standardisation activities and, finally, we highlighted those areas where analytical solutions are completely lacking thus requiring test development programmes. The results of this work will help standardisation bodies in their priority setting and can stimulate research activities to make reliable and relevant methods available for regulatory purposes.

## 2 Methodological approach

**Fig. 1:**
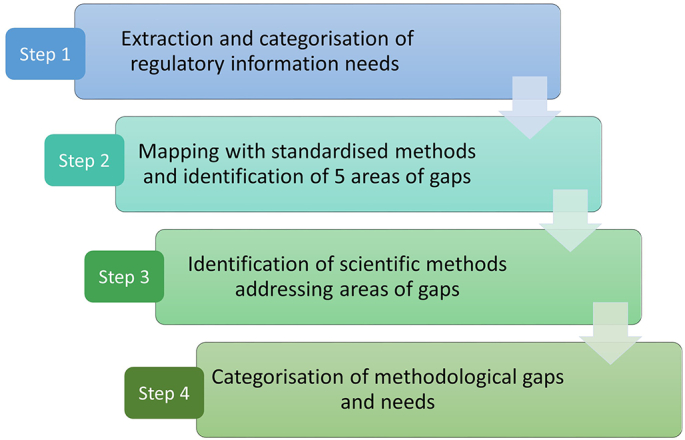
Main steps of the methodological gap analysis.

The gap analysis reported in this work was divided in four steps, as represented in Fig. 1.

### 2.1 Extraction of regulatory information needs (step 1)

In step 1, all the regulatory documents *e.g.* guidance documents, reflection papers addressing nanotechnology-based medicinal products and medical devices were compiled and categorised according to product class (*e.g.* liposomal products, iron-based colloidal products). Only documents published in English were considered. Regulatory information needs related to the quality and non-clinical safety assessment were extracted for each product class. When several documents were available for a specific class, all included parameters were collected. For the gap analysis, only parameters relevant for different product categories (at least three categories) or included in the regulatory documents addressing different product categories were considered.

### 2.2 Mapping with standardised methods (step 2)

Available standardised methods (ISO, ASTM International, CEN) were mapped against regulatory information needs identified in step 1. Endpoints for which no standardised methods are available were grouped into five broad areas of gaps.

### 2.3 Compilation of methods addressing areas of gaps (step 3)

Broad areas of gaps were broken down into more specific subcategories and endpoints as specified in regulatory documents or based on expert knowledge, and mapped against methods. Multiple sources of methods were considered including:

(i) standards (also, those under development) applicable to nanomaterials (ISO, ASTM International);
(ii) standards that are not specifically developed for nanomaterials, but used for pharmaceutical products and medical devices (ISO, pH. Eur.);
(iii) methods developed and optimized by research infrastructures and institutes with a strong expertise in nanomedicine, including the Nanotechnology Characterisation Laboratory (NCI-NCL)^1^ and the European Nanomedicine Characterisation Laboratory (EUNCL)^2^;
(iv) standard operating procedures and/or protocols established or under development by initiatives and research projects in the field of engineered nanomaterials and nanotechnology-based health products (*e.g.* FP7 Nanommune, NanoReg, H2020-REFINE);

Methods existing only as primary publication or proof-of-concept were generally not included in the analysis. However, where it was not possible to identify solutions based on mature methods, as a last resort, a literature search was performed to identify protocols that may have the potential to be considered for regulatory purposes, after assessing their accuracy and robustness.

### 2.4 Categorisation of methodological gaps and needs (step 4)

In step 4, the methods were evaluated according to their maturity level and proven applicability to nanomaterials or a nanotechnological platform, using scoring systems we developed for this goal (see Supplementary Material Tables). Based on this information, methodological needs in all subcategories related to five analysed areas of characterisation (see point 2.2): (i) surface properties, (ii) drug loading and release, (iii) kinetic properties in complex biological media, (iv) ADME parameters and (v) interaction with blood and the immune system, were classified into three categories: method optimisation, method validation/standardisation and method development. These categories of methodological needs are not mutually exclusive *i.e.* in some areas of characterisation more than one need could be identified.

## 3 Regulatory information needs for nanotechnology-based health products

Nanotechnology-based health products are regulated under the current regulatory frameworks for medicinal products and/or medical devices according to their mode of action. However, given their specific nanoscale-related properties additional characterisation needs were requested by the regulatory authorities in released guidance documents (Table S1). The U.S. Food and Drug Administration (FDA) have released a guidance document addressing the specific requirements associated to all drug products, including biological products, that contain nanomaterials [6]. For certain, more specific product classes, such as liposomes [[7], [8], [9]], polymeric micelles [10], iron-based colloidal products [11], and nucleic acid (siRNA)-loaded nanotechnology-based drug products [12], draft guidance documents and reflection papers have been provided by the FDA, the EMA and Japan's Ministry of Health, Labor and Welfare (MHLW) (Table S1). Those documents aim to describe specific properties of nanotechnological products that may affect the product quality, safety and efficacy. For medical devices containing nanomaterials, guidance to be followed have been provided by the Scientific Committee on Emerging and Newly Identified Health Risks (SCENIHR) and ISO [13,14]. The information needs, relevant for all classes of nanotechnology-based products (requested for at least three different product classes or included in FDA guidance for all drug products that contain nanomaterials) were grouped and are presented in Table 1 [15]. They are categorised into physicochemical and biological parameters, the latter including bioburden, pharmacokinetic and pharmacodynamic properties. Among the physicochemical parameters, a number of properties are related to drug delivery systems, which is the broadest application of nanotechnology-based medicinal products. The compiled information needs are not stand-alone requirements but should be considered in addition to applicable guidelines for medicinal products and medical devices. Therefore, most of them are specific to the nanotechnology field, or represent properties particularly relevant and/or challenging to assess in case of nanotechnology-based products (*e.g.*, stability, sterility).

**Table 1:** Regulatory information needs extracted from the regulatory documents addressing all classes of nanotechnology-based health products [15].

**Physicochemical parameters** **(if applicable)**	**Biological characterisation** **(if applicable)**
• Chemical composition ▪ Chemical structure ▪ Structural attributes that relate to function ▪ Crystal form ▪ Impurities ▪ Particle size and size distribution ▪ Shape and morphology ▪ Surface properties (*e.g.*, surface area, surface charge, chemical reactivity, ligands, hydrophobicity, and roughness); ▪ Particle concentration ▪ Porosity (if it relates to a function) ▪ Degradation path, kinetics and degradation products ▪ Stability, both physical and chemical under relevant conditions **Drug delivery systems** • Drug loading efficiency • Presence and distribution of any active ingredient associated with the nanomaterial and free in solution • Physical state of the active substance • *In vitro* drug substance /siRNA release rate in physiologically/clinically relevant media	**Bioburden control** ▪ Sterility and endotoxin levels **Pharmacokinetic parameters** ▪ Stability in blood and serum ▪ Biological fate ▪ Accumulation issues ▪ ADME ▪ Plasma protein binding (formation of protein corona over time) ▪ *In vivo* degradation/solubilisation rate and place of degradation **Pharmacodynamic parameters** ▪ Biocompatibility with blood and serum ▪ Additional risks associated with the exposure route: *e.g.*, haemocompatibility for iv administration ▪ *In vitro* uptake and cytotoxicity of nanomaterials to the phagocytes ▪ Interaction with enzymes ▪ Immunogenicity (ICH S8) ▪ Complement activation

## 4 Identification of major methodological gaps

To address the regulatory information needs summarised in Table 1, accurate, robust and validated methods that are fit for purpose need to be available. Criteria for the validation of analytical and bioanalytical procedures and for the regulatory acceptance of 3R testing approaches, including *in vitro* methods, have been provided by the EMA and ICH [[16], [17], [18]]. The required demonstration of method validity for a given purpose in a dossier submitted to competent authorities can be reduced if standardised and regulatory accepted methods are used. Therefore, availability of suitable standardised methods matching regulatory needs can reduce the resource burden and uncertainty for drug developers.

In the present analysis, existing standardised methods developed in the nanotechnology area were mapped against regulatory information needs reported in Table 1.

For physicochemical attributes, multiple methods have already been considered and standardised by the ISO Technical Committee 229 on Nanotechnologies and ASTM E56 committee. These methods can apply to products from different industrial sectors, including medical devices. Furthermore, a reference to an ISO method for particle size measurement is included in the European Pharmacopoeia (Ph. Eur.), extending its applicability to medicinal products. A review of available ISO and ASTM International standardised methods addressing nanotechnology-based products has recently been published [19], demonstrating that the majority of methods that could be relevant for health products are related to particle size, morphology and surface charge, while no standardised methods are available to assess other characteristics relevant for medical applications, *e.g.* drug loading and release kinetics.

Safety assessments are performed following ICH guidelines (medicinal products) and ISO guidance documents (medical devices) and currently, only few standardised methods are available to assess biological effects of nanotechnology-based products. The ISO guidance on the biological evaluation of medical devices contains a specific part dedicated to products containing nanomaterials (ISO/TR 10993–22:2017), but it provides only general considerations and not specific method protocol. In particular, characterisation of pharmacokinetic profiles of nanomedicinal products that can significantly differ from small-molecule drugs and assessment of immunological effects induced by such products needs suitable standardised methods to complement existing safety testing following ICH guidelines [20].

As a result of our analysis, five broad categories of characterisation were identified, for which no standard test method currently exist. Those categories include: (i) surface properties, (ii) drug loading and release, (iii) kinetic properties in biological media (including degradation and protein corona formation), (iv) ADME (absorption, distribution, metabolism and excretion) parameters and (v) interaction with blood and the immune system.

**Fig. 2:**
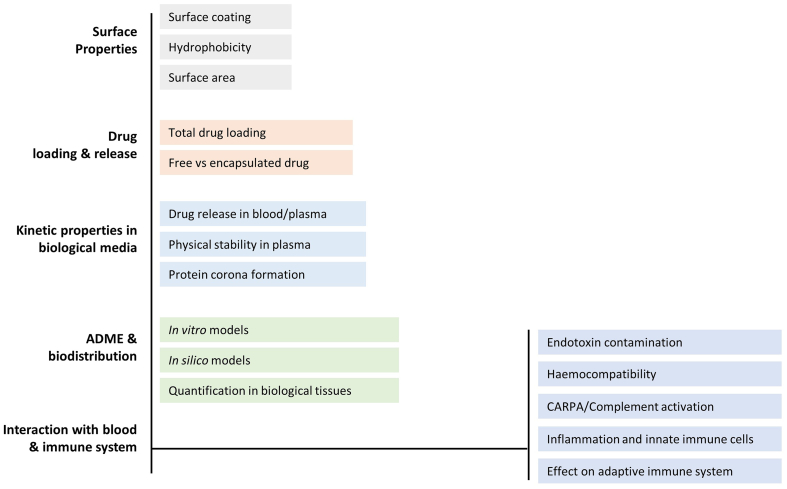
Categories and subcategories of methodological gaps, for which no standardised methods are available for nanotechnology-based health products.

In order to investigate more in-depth methodological needs, these broad categories of endpoints were divided into more specific subcategories (Fig. 2) and mapped against existing (not only standardised) methods that could provide the necessary information. The results of such exercise are presented in detail in the next section, category by category. Available methods matching regulatory endpoints are gathered in Supplementary Material, Tables S2-S4.

### 4.1 Surface properties

The surface of the nanoparticle determines the nature and the extent of its interactions with the external environment, *e.g.* with proteins and immune cells [21]. Surface properties, such as particle surface area, surface charge, chemical reactivity, ligands, and hydrophobicity are therefore critical material attributes that need to be measured and controlled. While ASTM and ISO standards exist for the analysis of the surface charge of nanomaterials [19], currently no standardised methods are available for the analysis of other properties, including hydrophobicity, quantification and homogeneity of surface functionalities and surface area (Table 2). However, promising methods and techniques exist and are described in the corresponding subsections.

**Table 2:** Overview on relevance and major challenges related to methods measuring surface properties with reference to regulatory documents.

**Area**	**Subcategories**	**Relevance**	**Challenges**	**Gaps**
Surface properties [[6], [7], [8], [9], [10], [11], [12], [13],^22^,^23^]	Coating analysis (amount, chemical composition, homogeneity)	Impact on the interaction with proteins, biomolecules, immune cells	Most of the existing methods are not standardised	– Limited technological solutions for soft nanomaterials, and/or for surface properties in solution– No methods for the heterogeneity of surface coatings
	Hydrophobicity			
	Surface area			

#### 4.1.1 Surface coating

Most nanomedicinal products possess a polymeric surface coating to enhance their dispersion, stability and biocompatibility. Polyethylene glycol (PEG) is by far the most used polymer for this purpose. Additional surface moieties can be included in the coating to enhance the therapeutic effects, *e.g.* to attempt active targeting or for other purposes. Ideally step-by-step, methods should be available to: (i) analyse the chemical nature of the surface coating (qualitative), (ii) quantify the amount of surface coating (quantitative), (iii) determine the homogeneity of the coating coverage and the conformational structure of its molecules.

In the case of inorganic NPs, multiple techniques are available to analyse the chemical composition of the coating and to quantify the total surface coverage (Table S2). For soft nanocarriers, *e.g.* lipid-based or polymeric NPs, chromatographic approaches can be developed on a case-by-case basis to identify and quantify components of the surface coating after particle dissolution [24]. The analytical methods are highly specific and must be tailored according to the chemical composition and physical properties of the NPs.

All the presented approaches are performed on NP samples that determine the average amount of the coating measured over a broad particle population. Measuring the coating heterogeneity, *e.g.* as variable number of ligands per nanocarriers or as a non-uniform covering of ligands on the particle surface is, to our knowledge, not possible with the existing technologies [25]. Attempts to measure heterogeneity in PEG coverage have been made at the basic research level by indirect measurements, *e.g.* by estimating particle roughness at the single particle level by using atomic force microscopy (AFM), or by fractionating particles by their hydrophobicity in hydrophobic interaction chromatography. However, those methods are not yet mature enough to be considered for regulatory purposes.

#### 4.1.2 Surface hydrophobicity

There has been only limited work aimed at developing reliable methods for surface hydrophobicity measurement applicable to nanomaterials, and currently no standards exist [26]. Standardised methods used for chemicals, *e.g.* water/octanol (K_ow_) partitioning method, are not applicable to nanomaterials [27]. An alternative to the K_ow_ method is usually the Contact Angle technique, which is only applicable to hard nanospheres, but not to soft NPs, such as liposomes, micelles, lipid-based NPs or polymeric NPs. Most of the alternative solutions are at the proof-of-concept stage, and to our knowledge they have not been validated [26]. The most mature method proposed by Valsesia et al. is based on measuring NP binding affinity to multiple collectors based on fluorinated hydrophobic surfaces with differential surface energy properties. The binding rate is calculated by measuring the number of NPs binding to the different collector as a function of time, by conventional dark-field microscopy [28]. This method is currently under consideration for standardisation within the OECD (Table S2).

#### 4.1.3 Surface area

Standardised method ISO 9277:2010 describes how to measure the specific surface area of nanoparticle powders by gas adsorption — the BET method (Table S2). However, this method is only applicable to inorganic NPs and even in this case, not reliable in the presence of a polymeric surface coating. This is reducing the range of applicability for nanomedicinal products. No standardised methods exist to measure the surface area of organic particles and of particles dispersed in aqueous suspension, which is the most representative state for the characterisation of medical applications. One method listed in the NanoReg Toolbox proposes to measure the wettable surface area of organic NPs in suspension by NMR, detecting the difference between the free and absorbed H-nuclear relaxation time [29,30]. However, its validation status is not known.

### 4.2 Drug loading and release (simple media)

Most of the nanomedicinal products on the market, in clinical trials or under development (*e.g.*, liposomes, emulsions, micelles, polymeric, and lipid-based nanoparticles) are drug delivery platforms consisting of two parts: (i) a pharmaceutically inactive nanocarrier and (ii) an active pharmaceutical ingredient (API), *i.e.* the drug substance(s), encapsulated in the nanocarrier. Encapsulation of APIs in advanced nanodrug delivery platforms helps to provide superior therapeutic efficacy and/or safety in comparison to the free API or legacy drug counterparts. The measurement of total drug loading in a nanomedicinal formulation is usually achievable by dissolution of the delivery vehicle (nanoparticle), using a surfactant or a suitable organic solvent, followed by the extraction and quantification of the API. More complex case-by case chemical approaches may be needed if the API is covalently bonded to the nanocarrier and must be released before its quantification. Quantification of the API is generally performed by liquid chromatography coupled to the most suitable detection systems, including UV-VIS, tandem molecular mass spectrometry (LC-MS/MS) or Inductively Coupled Plasma Mass Spectrometry (ICP-MS), as described in the protocols EUNCL-PCC-30 and NCI-NCL-PCC-14 (Table S2). These methods are generally able not only to quantify the API, but also to detect, identify and quantify possible impurities. The analytical methods are substance-specific and cannot be generically standardised. Major challenges in the field are related to nanocarriers encapsulating emerging classes of APIs, like large nucleic acids, including DNA or mRNAs, where techniques for the API identification, quantification and detection of impurities are still under development (Table 3) [31].

**Table 3:** Overview of relevance and major challenges related to measurement of drug loading and release with reference to regulatory documents

**Area**	**Subcategory**	**Relevance**	**Challenges**	**Gaps**
Drug loading & release [[6], [7], [8], [9], [10], [11], [12]]	1) Total drug loading	Impact on efficacy safety Monitoring of API leakage during storage and use (stability)	• Need to adapt the methods to each nanocarrier/API combination • Separation of API from nanocarrier prior to API quantification (subcategory 2) can induce artefacts	detection and quantification of large API such as nucleic acids
	2) Free *vs* encapsulated drug			

The measurement of free API (percentage of the total API content encapsulated into nanoparticle) and of drug release is a two-step process, depending on the availability of analytical methods that can accurately separate the carrier from the free API prior to its quantification. Most of the analytical methods currently available for the separation of the free API from the nanocarrier are adapted from techniques conventionally used for bioanalytical purification of nano-formulations, including chromatographic methods, liquid-liquid extraction and equilibrium methods. The method chosen should be able to separate the medium with the free drug from the particles (i) without affecting the carrier integrity, (ii) without inducing leakage of drug by other means (*e.g.* dilution) and (iii) without influencing the concentration equilibrium of the drug between the encapsulated and the free state. EUNCL and NCI-NCL have developed and validated protocols for separation of free *vs* encapsulated drugs by ultrafiltration that are applicable to multiple nano-formulations encapsulating classical small drugs (Table S2). Many other protocols are available in the literature that are tailored to specific nanomedicine classes using other separation techniques, such as the use of Solid Phase Extraction for lipid-based nanoparticles [32]. In case of extremely challenging products, the use of complementary methods could be used to obtain an orthogonal confirmation of the results.

### 4.3 Kinetic properties in biological media

In contrast to classical drugs where stability studies in conditions mimicking the physiological exposure are only focalised on the chemical stability, nanomedicinal products require the evaluation of the stability in biological media considering three additional aspects: (i) the release kinetics of the API from the carrier in the presence of the plasma proteins, (ii) the physical stability of the nanocarrier regarding the change in size and polydispersity of the particles following their contact with plasma proteins and (iii) the adsorption of plasma proteins onto the particle surface leading to the formation of a protein corona altering the surface properties (Table 4). Moreover, analysing the chemical stability of complex drug products, *e.g.* towards oxidation and degradation of lipid-based NPs containing polydisperse components, by standard analytical chromatography approaches may be technically very demanding. Molecular stability can be affected by the biological media, *e.g.* by chemical or enzymatic hydrolysis of labile structures like ester bonds. Those aspects introduce critical methodological challenges, as described in the following paragraphs.

**Table 4:** Overview of relevance and major challenges regarding the of kinetic properties in biological media with reference to regulatory documents.

**Area**	**Subcategory**	**Relevance**	**Challenges**	**Gaps**
Kinetic properties in biological media	Drug release in blood/ plasma [[6], [7], [8], [9], [10], [11], [12]]	Impact on the therapeutic efficacy and safety	Separation and quantification of encapsulated and unencapsulated drug fractions	Measurement of drug release (large APIs)
	Physical stability (Size changes) [[6], [7], [8], [9], [10],^13^]	Product stability in human body	Separation of particles from the blood proteins	No technological solutions for small “soft” organic particles
	Protein corona formation (amount and identification of bound proteins) [6,[8], [9], [10],^13^]	Impact on the interaction with immune system, safety and efficacy	Separation of NP–protein corona complexes from excess plasma Standardisation of plasma composition	No technological solutions for small organic particles

#### 4.3.1 Drug release in blood/plasma

Once a nanocarrier-drug delivery system is injected intravenously, the existence and fate of: (i) the API encapsulated (*e.g.*, bound) in the nanocarrier, (ii) the free API and (iii) the API bound to plasma proteins should be evaluated, since the three species may have very different pharmacokinetic profiles. From a technical point of view, measuring the dynamic tissue distribution of all the fractions is a very challenging task, due to the complexity of separating and quantifying encapsulated and unencapsulated drug fractions in blood and tissues.

Despite the lack of standardised methods to perform such measurements, a relevant method developed by NCI-NCL (Table S2, NCL PHA-2) is now under evaluation for standardisation by the ASTM E56 committee [33]. This assay utilises an improved ultrafiltration method for nanomedicine fractionation in plasma, based on the use of a stable isotope analogue of the API, spiked into a plasma sample containing the nanomedicine under investigation in order to (i) precisely measure the degree of API bound to plasma protein, in addition to the unencapsulated, and encapsulated API fractions, and (ii) take into account all sample loss during the process, thus correcting artefacts. No solutions currently exist for the reliable quantification of large APIs (*e.g.* mRNA, DNA), or for cases where at least one stable isotope analogue of the drug is not available.

#### 4.3.2 Physical stability in biological media (particle size)

The ionic strength, the proteins and the enzymes in blood and plasma can impact the physical stability of a nanomedicinal formulation, *e.g.* by inducing aggregation/agglomeration or by enhancing particle dissolution (dramatic size changes). Even if particles are stable in complex biological media, they interact with plasma proteins; indeed, protein binding on the particle surface generates an extra layer, the “protein corona”. EUNCL and NCI-NCL laboratories have jointly developed multiple protocols for size measurements, that have been tested in complex biological media under specific conditions and suggest to use them in a step-by-step approach of incremental complexity. The most widely used sizing technique, batch mode dynamic light scattering, can be used as first check to investigate major size changes, *e.g.* fast aggregation or particle dissolution in serum or plasma. High resolution techniques such as particle tracking analysis (PTA), analytical ultracentrifugation (AUC) and asymmetric flow field flow fractionation (AF4) coupled to sizing detectors may be used in a second step to increase the measurement resolution, *e.g.* to detect small size changes like the formation of the protein corona (Table S2). Among the listed high resolution techniques, AF4 was shown to be the most promising method to provide accurate size information for polydisperse samples in physiological media and for protein binding studies by fractionating the free protein and the NPs in complex media prior to performing the sizing measurements [34]. Due to the technical challenge to separate proteins from particles of comparable size and density, no technical solutions currently exist to measure size changes of small soft organic particles in plasma, *e.g.* dendrimers or small polymeric micelles possessing an average size below 20–30 nm (diameter).

#### 4.3.3 Protein corona formation

Nanomaterials entering the blood circulation interact with proteins, sugars, and lipids, resulting in the formation of a protein corona, which influences the host biological response, thus affecting processes such as particle uptake by phagocytic cells and biodistribution.

There are two main approaches to study the protein corona: (i) quantification of the total amount of proteins binding to the particles' surface and (ii) identification of individual corona proteins separated by gel electrophoresis and determined by mass spectrometry (proteomics). The easiest and more mature method aims to quantify the total amount of proteins binding to the NP by two-dimensional polyacrylamide gel electrophoresis (2D PAGE) (Table S2). Individual proteins can be identified by mass spectrometry, either following 2D PAGE or by separate proteomic approaches [35].

As reported by Monopoli et al. [36] there are a few critical steps to take into account for the corona analysis, including (i) biological fluid (plasma or serum) collection and harvesting, (ii) NP incubation with biological fluid and (iii) separation of NP–protein corona complexes from excess plasma to remove loosely bound and free proteins. From a technical perspective (point iii), the application of corona quantification/composition analysis is limited to particles that can be separated from plasma by centrifugation (*e.g.*, metal oxides, metal colloids, core-shell materials, large polymeric NPs). No technological solutions currently exist for small organic nanocarrier systems (Table 4).

Another important open question for the community is how to first define, and then standardise the composition of the biological media to be used for corona studies, in order to mimic the real plasma composition in the specific clinical application, and to assure comparability between different studies. This challenge needs to be addressed before the relevance of protein corona studies for safety and efficacy prediction can be demonstrated.

Finally, slightly different challenge is related to the evaluation and monitoring of the quality including the physicochemical stability during the product shipment and storage. Majority of the methods described above are dependent on specialized infrastructure and analytical competence limiting their use in the clinical setting. This becomes an issue of particular relevance for complex pharmaceutical entities like protein therapeutics [37] and nanotechnology-based health products, and has recently been brought to global attention in the case of nanoformulated mRNA-based vaccines against Covid-19, where strict cold-chain requirements pose a significant challenge for their widespread application *e.g.* in many developing countries.

### 4.4 ADME and biodistribution

To understand the absorption, distribution, metabolism and excretion (ADME) profile of a product under investigation it is important to use a range of model systems from *in vitro* cell-based models to *in vivo* animal models. In the early stages of development, the use of human subjects is not feasible. However, the more recent evolution in experimental and computational platforms including mathematical modelling can support an integrated evaluation of biodistribution processes. Major methodological challenges related to this section are summarised in Table 5.

**Table 5:** Summary of challenges and gaps for the ADME and biodistribution methods with reference to regulatory documents

**Area**	**Subcategory**	**Challenges**	**Gaps**
ADME and biodistribution [[6], [7], [8], [9], [10], [11], [12], [13],^22^,^23^]	Detection and quantification in biological matrices	Need for method adaptation for each type of nanomaterials	Detection and quantification of intact nanoparticles except for fluorescent labelled or metal nanoparticles.
	*In vitro* models	Relevance for human biodistribution unknown	Advanced cell models including specialized cell types
	*In vivo* models	Inter-species variation between animal models and humans	Suitable tumour models with vascularization comparable to human cancer
	*In silico* modelling	– Validation and optimization for each platform– Lack of physiological data to feed the models	

#### 4.4.1 Detection and quantification in biological matrices

All assays require detection and quantitation of nanomaterials in biological matrices, such as tissues, cells and subcellular structures. This can be in the form of either detecting the API, which is encapsulated or conjugated to a nanocarrier or, in some cases, involve detection and quantitation of the whole nanoparticle itself. In the case of detecting the API, bioanalytical methods such as LC-MS/MS, ICP-MS and UV-spectroscopy (Tables S2 and S3) can be used but must be coupled to chemical or physical extraction of the API from the nanocarrier (see also section 4.2). Whole nanoparticle detection can be more complicated and might only be relevant for a certain subset of nanotechnological platforms [38]. This can include nanoparticles which are natively fluorescent (such as some inorganic, metal NPs), or can be loaded with fluorescent markers for detection by flow cytometry. Various imaging techniques can be used in the case of labelled NPs or occasionally bioanalytical methods such as ICP-MS which can be used to detect intact metal nanoparticles [39] (Box 1).

Box 1 **Available techniques for NPs detection in cells**

• Flow cytometry /Fluorescence microscopy (fluorescently labelled NPs)
• ELISA (biotinylated NPs)
• Molecular imaging (radiolabelled NPs)
• Time-lapse video microscopy
• Electron microscopy (solid-core NPs)
• ICP-MS (metal NPs)

Alt-text: Box 1

#### 4.4.2 In vitro models

While many small-molecule therapeutic agents are administered orally, the vast majority of nanotechnology-based health products that have reached the clinic to date, have low oral bioavailability. Despite this, *in vitro* assays assessing the gut absorption of nanomaterials are widely used, including the ubiquitous Caco-2 permeability assay (Table S3). The Caco-2 cell line is an immortalised cancer cell line, which forms a polarised monolayer when cultured on transwell plates for extended periods. To establish further physiological relevance the Caco-2 cell line is combined with other cell lines in co-culture transwell systems to better model the influence of specialized intestinal cells such as gut resident immune cells and mucus producing goblet cells [40]. Other *in vitro* models are also available, based on primary cells or immortalised cell lines (Table S3), however their use is mostly limited to the research area. While some of the issues of variability might be mitigated by the use of cell lines, many *in vitro* assays rely on the use of immortalised cell lines, which are often cancer, or cancer hybrid lines and which can be vastly different from normal human cells.

In addition, understanding the intracellular trafficking of nanomedicinal formulations might have value for determining whether cell uptake completely prevents bioavailability of the encapsulated molecules (in the case of degradation, or sequestration) or simply delays it (if the formulation is subsequently released from the cell). Assays to assess this are complex and currently not widely used in the determination of biodistribution of nanomedicinal products.

#### 4.4.3 In vivo ADME

It is well established that nanotechnology-based products have a certain propensity for accumulation in tissues such as the liver and spleen which can prevent their distribution to the target tissue [41]. Mouse and rat models are used extensively to track the distribution of nanomaterials in target tissues as well as blood plasma and urine samples. Methods are similar to those used for small-molecules where animal models are treated with titrated doses over considered time points before the organs are harvested and processed for extraction of the API or whole nanoparticle. This can include tissue homogenisation, or sectioning to track labelled NPs visually. To date, the vast majority of clinically approved nanomedicinal products are accepted for use in oncology. However, there is evidence to suggest that xenograft models used to assess oncology therapeutics have more highly vascularised tumours, compared to human cancer, and therefore are not optimal to predict the pharmacokinetic profile in patients (Table 5).

#### 4.4.4 In silico computational modelling

Computational tools for the assessment of biodistribution of nanotechnology-based health products are potentially very valuable due to their ability to simulate complex biological systems and the pharmacokinetics of the administered formulation through those systems. In particular, physiologically based pharmacokinetic (PBPK) modelling allows for the development of a complex, full body mathematical model, which can describe both physicochemical properties of the pharmaceutical compound, and human physiological processes. In PBPK modelling, each relevant organ is created as an individual compartment within the model, described by ordinary differential equations (ODE) which define the behaviour of that organ. Model parameterisation makes use of physiologically relevant data, derived from *in vivo*, *in vitro* or clinical analysis, in order to tailor the model to the specific nanomedicinal formulation. While the PBPK model does rely on physiological data, and is frequently hindered by the lack of such data, longer term goals for PBPK modelling aim to reduce the use of animal models by incorporating *in vitro* data into a human model. Application of PBPK modelling to the field of nanotechnology is relatively new, around 20 models of organs (the majority using a mouse/rat model) considered most relevant for nanomaterial distribution have been described in the literature (Table S3). However, these require more validation before being adopted for regulatory purposes.

### 4.5 Interaction with blood and the immune system

Interaction of nanomaterials with the immune system has been frequently reported in the scientific literature [42,43] demonstrating the capacity to activate the immune system or, in some cases, suppress/reduce the immune response. In the case of nanomedicinal products such reactions can lead to adverse effects or increased clearing from the body resulting in reduced therapeutic efficacy of the product. The assessment of the immune response to nanotechnology-based products has been recommended in regulatory documents addressing several nanotechnology-based product classes (Table 6). Starting from regulatory endpoints specified in those documents and expanding them to endpoints routinely tested in the immunotoxicity assessment of pharmaceuticals [44] and medical devices [45], we identified subcategories of the immune effects, that are described in the following subsections. The major methodological challenges and gaps related to different subcategories are gathered in Table 6. Considering that inter-species variations are particularly evident for the immune system, we have not considered animal tests but focused selectively on *in vitro* test methods (Table S4) that could provide relevant information. Most of these methods have specifically been designed or verified for the testing of nanomaterials, since the use of conventional methods can be hampered by interference of nanomaterials with assay components or readouts [46].

**Table 6:** Summary of methodological challenges in the area of interaction with blood and the immune system with reference to regulatory documents.

**Subcategory**	**Specific pathways/ Endpoints**	**Tested Adversity**	**Challenges and gaps**
Endotoxin contamination [6,8,9,14]	Quantification of endotoxin and pyrogens	Microbiological contamination	• Detection of encapsulated endotoxin • Quantification of uptake by phagocytes (unlabelled organic nanomaterials) • Interference of nanomaterials with commonly used readouts (fluorescence, absorbance, chemiluminescence) • Lack of advanced *in vitro* systems that could include interactions between different immune cell types • Relevance of *in vitro* assays for the effects in humans • Variability of results (whole blood, plasma, primary cells)
Haemocompatibility [6,10,12,13]	Red blood cells lysis Platelet aggregation, Coagulation	Haemolysis Thrombogenicity	
CARPA and complement activation [7,9,10,12,13]	Complement activation Activation of secretory cells	Hypersensitivity reactions/CARPA	
Inflammation and innate immune cells [6,7,9,[11], [12], [13],^22^,^44^]	Inflammasome activation Macrophage function NP uptake by phagocytes NK cell activity	Inflammation Increased clearance from the body Modulation of immune response	
Effect on adaptive immune system [10,13,14,44]	Dendritic cell maturation Effects on lymphocytes Antibodies production	Immunosuppression Increased clearance from the body	

#### 4.5.1 Endotoxin contamination

Endotoxins are lipopolysaccharides (LPS) which are components of the outer wall of gram-negative bacteria. LPS contamination may occur in any step of manufacturing and handling of nanotechnology-based products and lead to adverse effects. Therefore, for medicinal products exposure limits have been set. Their contamination may also affect the outcome of toxicological assays. Hence, the potential presence of LPS in the testing sample should be determined prior to performing such assays.

The most commonly used assay for *in vitro* endotoxin determination is the Limulus Amoebocyte Lysate (LAL) assay. Three types of the LAL assay exist: the turbidimetric, the chromogenic, and the gel clot assay. While the LAL is a reference for endotoxin determination, included in the Ph.Eur. and ISO standards (Table S3), interference has been observed testing conventional medicines and nanomaterials. The extent of interference of nanomaterials depends on different aspects, including the bacterial strain as source of LPS, the LPS concentration, the type of the assay and physicochemical properties of the nanomaterials. Therefore, special precautions and assay optimisation are necessary when using LAL-based assays to test nanotechnology-based products. Another challenge is related to the detection of endotoxins which are encapsulated inside a nanocarrier thus giving false negative results [47,48].

Testing for endotoxins can also be performed by testing for pyrogenicity. An *in vitro* pyrogen assay is the monocyte activation test (MAT), where the presence of pyrogens results in the production of proinflammatory cytokines by human monocytes. The MAT is a standardised method, included in the Ph.Eur., which can detect the pyrogen, even if it is encapsulated (Table S4), but does not distinguish between general pyrogens and endotoxins, specifically. Alternatives to LAL-based methods need to be developed for nanotechnology-based products. Some alternative approaches exist, based on TLR4 reporter cells [49] or measuring endotoxin indirectly *via* 3-hydroxylated fatty acids of lipid-A [50], however they are still in early stages of development (Table S4).

#### 4.5.2 Haemocompatibility

**Fig. 3:**
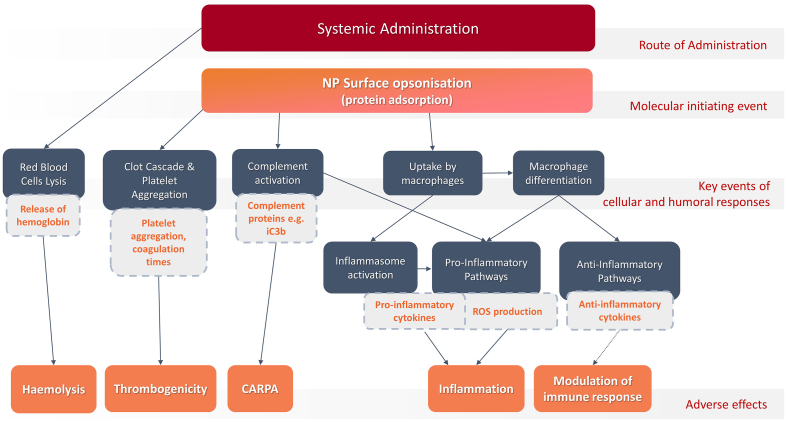
Schematic illustration of the main (non-exhaustive) cellular responses and pathways (dark blue boxes) leading to adverse effects of nanotechnology-based health products following systematic administration, and corresponding *in vitro* endpoints of biological assays (light blue boxes). NP: Nanoparticle. (For interpretation of the references to colour in this figure legend, the reader is referred to the web version of this article.)

Assessment of haemocompatibility, including effects on red blood cells (haemolysis) and thrombogenicity potential is required for medicinal products and medical devices that will be in contact with blood (Fig. 3). Haemolysis refers to the damage to red blood cells, which may lead to anaemia and other life-threatening conditions. The *in vitro* haemolysis assay was found to be highly predictive for *in vivo* studies identifying haemolytic and pro-inflammatory potential of nanoparticles [51]. The ASTM International protocol E2524–08 (Table S4) to study nanoparticle haemolytic properties sets a threshold for *in vitro* haemolysis at 2% of the positive control. This method is referenced in the ISO guidance for medical devices containing nanomaterial (ISO 10993-22).

The guidance also requires the evaluation of the risks of thrombogenicity involving one or more components of the blood coagulation system. The mechanisms of thrombogenicity are often complex and involve multiple cell types (thrombocytes, leukocytes, endothelial cells) and plasma coagulation factors (Fig. 3). As such, there is no single assay that can be used to assess nanomaterial thrombogenic potential. Nevertheless, *in vitro* assays targeting platelets and three plasma coagulation pathways: extrinsic, intrinsic (also known as contact activation pathway) and common, have been developed; they are used for estimating nanoparticle pro- and anti-coagulant properties. Protocols based on the measurement of platelet aggregation have been developed by NCI-NCL and EUNCL laboratories (Table S4) and are good candidates for standardisation. Other methods evaluating interaction of nanomaterials with platelets and the clotting system are available in the scientific community [52,53], but require more validation before becoming adoptable as regulatory standards.

#### 4.5.3 CARPA and complement activation

**Fig. 4:**
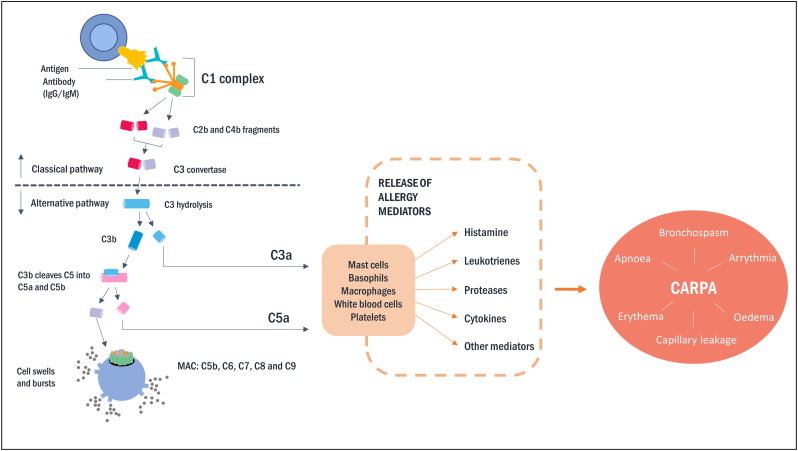
Main steps of complement system activation leading to complement activation-related pseudo-allergy (CARPA). In the classical pathway, mainly IgG or IgM bind to structures on the surface of NPs resulting in cleavage of C3 into C3a and C3b, while the alternative pathway requires C3 on the surface and can itself convert C3 to C3a and C3b. C3a and C3b induce inflammation, and C3b induces Membrane Attack Complex (MAC; C5b—C9) formation. Anaphylatoxins C3a and C5a induce activation of mast cells, basophils and leucocytes secreting allergy mediators leading to CARPA symptoms.

Complement activation-related pseudo-allergy (CARPA) is a hypersensitivity reaction, characterised by mild to severe cardio- pulmonary symptoms and reported in the context of nanotechnology-based health products, in particular liposomes [54,55]. This anaphylactic reaction is not IgE-mediated but triggered by complement activation (Fig. 4). Therefore, the activation of the complement system *in vitro* is the most used approach to evaluate the risk of CARPA in patients.

The complement system can be activated *via* the classical, lectin or alternative pathway. The latter is not only the predominant one in health and disease, but also critical in complement activation by many nanomedicinal products [[56], [57], [58]]. The information on the involved pathway can be obtained depending on the selected component involved in the cascade, though, robust assays allowing precise identification of a specific pathway are lacking. Most advanced current approaches are based on the quantification of iC3b, which is generated following C3 activation in any of the three pathways. C3b and iC3b prime the surface of a nanoparticle for engulfment by leukocytes and macrophages through complement receptors. A method based on iC3b measurement in human plasma is currently in the standardisation process by ASTM International (Table S3). Another active standard by ASTM International provides a method to test for whole complement activation in human serum by medical devices coming into contact with blood. However, its applicability to nanotechnology-based products is not known. In this case additional considerations and controls might be applicable.

Additional approaches to measure complement activation consider deposition on the surface of NPs of complement factors [59] or denatured proteins, and their recognition by natural antibodies [60]. In addition, the protein corona (see section 4.3) plays a significant role in complement activation as C3b binds to proteins, dissociates together with the soft corona, and “new” C3 may bind to the nanoparticle [61]. The exchangeable nature of the protein corona could lead to continuous shedding of complement factors and re-opsonisation *in vivo*.

Assessment of the impact on cells expressing anaphylatoxin receptors, such as basophils or mast cells can complement information on the mechanism leading to CARPA. Upon activation, these cells are able to release the content of granules as an early-phase response (histamine, serotonin), and a range of mediators as a late-phase response (prostaglandins and leukotrienes) leading to cardio-pulmonary symptoms (Fig. 4). A method evaluating the effect of nanotechnology-based products on the activation of basophils is currently in early-stage development in the REFINE project (Table S4).

#### 4.5.4 Inflammation and innate immune cells

One of the first forms of defence employed by the innate immune response is accomplished by pattern recognition receptors (PRRs) encoded in the germline to recognise pathogen-associated molecular patterns (PAMPs). These receptors may be either on the membrane of many cell types *e.g.* Toll like receptors (TLRs) or inside their cytoplasm *e.g.* Nod-like receptors (NLRs). Among the NLRs are the inflammasomes, of which the NLRP3 inflammasome is the most fully characterised one. Upon activation, NLRP3 recruits the adapter protein ASC, involved in the activation of caspase-1. Caspase-1 processes pro-IL-1β and pro-IL-18 to their biologically active forms. Activation of the NLRP3 inflammasome is a widely studied effect of nanotechnology-based products [[62], [63], [64]]. Available methods are based on the measurement of corresponding pro-inflammatory cytokines, chemokines and chemoattractant capacity (Table S4). A method evaluating the effect of nanotechnology-based products on inflammasome activation is currently in early-stage development in the REFINE project (Table S4).

In addition, the effect on macrophage function and uptake by phagocytotic cells can provide relevant information not only on proinflammatory potential of nanotechnology-based products but also on their pharmacokinetic properties and distribution in the body. Macrophages are one of the most important parts of the innate immune system that recognise, engulf and destroy pathogens, foreign particles and damaged or dead cells. Macrophages can differentiate into (at least) two subtypes, the pro-inflammatory (M1) type and the pro-fibrotic/anti-inflammatory M2 type. M1 macrophages are phagocytic, playing a major role in host defence, while M2 macrophages are involved in tissue remodelling and diseases such as fibrosis. An improved foreign body reaction(less fibrosis, more vascularisation) in response to implanted medical devices was found to be associated with a local shift from M2 to M1 macrophages [65]. A method evaluating the effect of nanomaterials on M1 and M2 macrophages, isolated from human blood is currently being developed in the context of the REFINE project (Table S4). Other approaches are investigating the macrophage capacity to produce Reactive Oxygen Species (ROS) and to engulf apoptotic cells [66] (Table S4). Several methods were developed by the European project Nanommune and are included in the Nanommune Quality Handbook [67]. In addition, an ISO standardised method for assessing the generation of nanoparticle-induced ROS in a murine macrophage cell line is available. However, the use of appropriate controls for interference is required, as many nanomaterials can interfere with commonly used readouts such as fluorescence or absorbance measurements. Moreover, the use of non-human cells makes extrapolation of the results to humans even more difficult.

Phagocytes are the type of white blood cells (mainly macrophages and neutrophils) that have the ability to phagocytose bacteria, foreign particles and dying cells, to protect the body. Evaluation of NP uptake by phagocytes is facing technological challenges related to the detection and quantification of NPs in biological structures (see section 4.4). Available methods often have material-dependent applicability, and in many cases pre-labelling of NPs is required prior to the use of specific readouts. A method based on luminol chemiluminescence, activated by the low pH of phagolysosomes, is currently in the process of standardisation by ASTM International (Table S4).

#### 4.5.5 Effect on adaptive immune response

Effects on the adaptive immune response can (virtually) only be evaluated *in vivo*. The assay that is nowadays considered the “gold standard” for effects of compounds and drugs on the adaptive immune system is the T-cell dependent antibody response (TDAR). Next to the antibody levels, in the same animals, also effects on cellularity (cell number) and immune cell subsets can be evaluated. The latter is done by immunophenotyping, nowadays most often performed by FACS. A new development is the pursuit of an *in vitro* replacement of the TDAR, the Human Leukocyte (HuLa) assay (Table S4), which showed some promising results on nanomaterials [68]. However, the complexity and intricacy of a primary immune response cannot be mimicked by currently available *in vitro* methods or array of methods. Rather, effects on some of the individual parts of such a response can be evaluated *in vitro*, being effects on dendritic cells maturation, T-cell proliferation and B-cell proliferation (Table S4). Much more sophisticated *in vitro* models would be necessary to evaluate interactions between different immune cell types, and with their surrounding tissue.

#### 4.5.6 *In vitro* models for immunotoxicity testing

The application of *in vitro* testing, for assessing the immunological and haematological interactions of novel therapeutics has been in place for some time [69] though is yet to be completely accepted as part of a regulatory protocol [20]. There are a number of advantages to the use of *in vitro* assays to assess immunotoxicity including, but not limited to: higher throughput assessment of materials or cell sources, exploration of possible mechanisms behind observed immune stimulation or suppression, reduction in the use of preclinical species and closer relevance to the intended human population [70]. However, current *in vitro* methods require more validation/standardisation and many of them have drawbacks in their relevance/application to possible *in vivo* interactions in humans (Table 6). The immune system is complex and can, broadly, be divided into innate (functioning without prior antigen exposure) and adaptive immune responses. Cells of the innate immune system express a plethora of pattern recognition receptors that have been shown to bind to NPs in order to bring about an immunological response. However, this complexity can make determination of the precise interaction of nanotechnology-based products with relevant immunological components difficult to achieve.

In terms of result reproducibility, procurement possibilities and safety, the use of cell lines can offer advantages over primary cells. However, some immunological functions cannot or can only poorly be reproduced by a cell line. The use of whole blood, or its protein and cellular composites, is a powerful tool in the understanding of nano-immune interactions and is a step closer to the *in vivo* environment that materials may encounter when used in humans. However, there is significant inter-individual variability in immunological responses to foreign organisms and material [71], which, in turn, may extend to their potential response to nanomaterials. In addition to the individual's health status there are also immunological variations caused by the individual's genetic make-up, individual's age, as well as seasonal and circadian factors as a result of differences in cell populations, cytokine responses and serum proteins [71].

At present, there is no, regular, provision for the inclusion of these factors in routine immunotoxicological testing but should be considered as a factor for future analysis. This may also be borne out when considering the translatability of these *in vitro* assays to *in vivo* readouts. The correlation between *in vitro* and *in vivo* immunotoxicity assays has been reviewed elsewhere but a number of *in vitro* immunotoxicity assays have been shown to have good (haemolysis, complement activation, cytokine secretion and phagocytosis) or fair (thrombogenicity, leukocyte proliferation and colony forming unit capacity) correlation [72]. These *in vitro* assays also help to identify the mechanism by which NPs may be causing the observed effect; however, each assessment is in isolation and does not, necessarily, reflect how each of these systems may interact *in vivo*. Complement activation may be assessed using human plasma samples, however, often only certain proteins are measured in isolation of the broader cascade. In addition, the effect on cells expressing anaphylatoxins [73], and the modulation by cytokines [74,75] would require examination of how these complex processes may fit together in more complex cell, or co-culture, systems. Finally, depending on the route of administration, the immune cells that nanoparticles may encounter may vary. Although some overlap is envisaged, it is important to consider the context of each application route and prioritise assessment of the cells present.

## 5 Summary of methodological needs and gaps

By analysing available methods applicable to nanotechnology-based health products, we confirmed insufficiency of methods in five areas related to physicochemical characterisation (PCC), biodistribution and interaction with blood and the immune system. Moreover, we have identified more specific methodological needs such as nanomaterial-dependent adaptation of methods (category 1), validation and standardisation of methods that are in early stages of development (category 2) and finally, development of additional methods, in those areas where no or very few methods are currently available (category 3) (Table 7).

**Table 7:** Categorisation of main methodological needs.

**Category 1** **Method adaption to specific/new nanomaterial**	**Category 2** **Method validation and standardisation**	**Category 3** **Development of additional** **methods**
**PCC:** All PCC methods have to be optimized for each specific NP/API class, according to general guidelines.	**PCC:** ➢ Drug loading and drug release in complex media ➢ Hydrophobicity ➢ Physical stability in complex media	**PCC:** ➢ Release and quantification of large API such as nucleic acids ➢ Specific surface area evaluation in aqueous media ➢ Quantification of surface coating and analysis of coating heterogeneity ➢ For small organic nanomaterials: Fractionation methods for stability studies in complex media and determination of protein corona composition
**Biodistribution and ADME:** Adjustments are necessary for each technological platform, for ADME and for *in silico* models		
	**Biodistribution and ADME:** ➢ Barrier models *in vitro* ➢ Detection/quantitation of whole nanomaterials in simple and complex media including in cells ➢ Existing PBPK models	
**Immune system:** ➢ LAL-based methods for endotoxin		
		**Biodistribution and ADME:** ➢ Detection/quantitation of unlabelled organic nanomaterials in cells, tissues and subcellular structures ➢ Sophisticated *in vitro* models for the prediction of human pharmacokinetics
	**Immune system:** ➢ Effect on macrophages ➢ Uptake by phagocytes ➢ Inflammation ➢ Activation of complement system ➢ Thrombogenicity ➢ Effect on lymphocytes and antibodies (existing methods)	
		**Immune system:** ➢ Endotoxin contamination: alternative methods to LAL ➢ Advanced *in vitro* models to assess effects on adaptive immune system

**Fig. 5:**
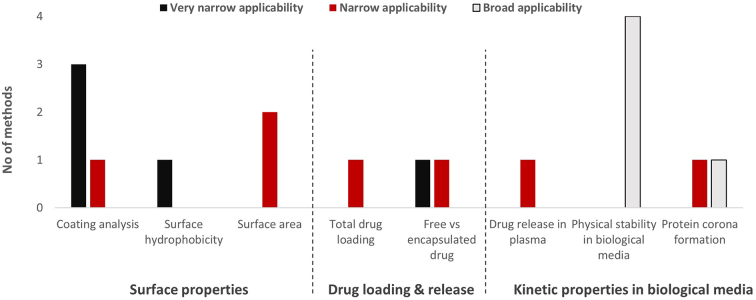
Overview on applicability of methods in three areas of physicochemical characterisation, including surface properties, drug loading and release and kinetic properties in biological media without considering the status of method validation. Very narrow applicability: methods applicable to one specific nanomaterial or nanomaterial type (*e.g.* AuNPs, liposomes); Narrow applicability: methods applicable to multiple nanomaterial types (*e.g.* inorganic or organic), Broad applicability: methods applicable (eventually with some specific limitations, *e.g.* particle size) to all nanomaterial types. Methods represented in the figure are those listed in the Supplementary Material Table S2.

The main challenge for methods addressing PCC characterisation is their high level of specificity yielding applicability to certain type of nanomaterials only (Fig. 5). While several methods are available for metallic and specifically gold NPs (Table S2) for the analysis of surface properties, no or only very few technological solutions exist for other types of nanotechnological platforms, in particular small organic nanomaterials. In some cases, the method adaptation and optimisation can extend the applicability to additional nanotechnological platforms. Such adaptation is also required in case of *in silico* models and toxicological methods developed for small-molecule drugs (such as endotoxin quantification) that might require special considerations and additional controls when used with nanomaterials (category 1, Table 7).

**Fig. 6:**
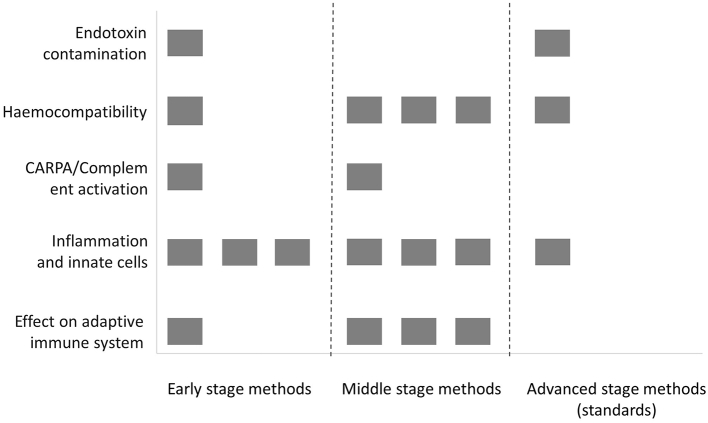
Overview on the status of validation of available *in vitro* methods addressing interaction of nanotechnology-based products with blood and the immune system. Early-stage methods: methods existing as primary publications or in regular use in research, but not commercially available, Middle stage methods: used routinely in relevant R&D environment, with assessed intra-lab or inter-lab variability; Advanced stage methods: standardised test methods (ASTM International, ISO). Methods from outside the nanotechnology field are not included. Methods represented in the figure are those listed in the Supplementary Material Table S4.

Another need is related to method validation and standardisation. Most of the methods coming from the scientific community and covered in this review are still under development and require more validation in terms of inter-laboratory repeatability and reproducibility. This includes several methods for the PCC characterisation in biological media such as physical stability, drug loading and drug release, as well as existing tools for the characterisation of the particle surface. This category also covers the majority of *in vitro* immune methods addressing haemocompatibility, effect on innate immune cells and inflammation, where a number of test methods are in early or middle stage of development (Fig. 6). More advanced methods addressing *e.g.* surface hydrophobicity, complement activation and uptake by phagocytes, for which the potential has been recognised, have already entered the standardisation process. In the area of biodistribution, existing approaches for the quantification of nanomaterials in cells and tissues, as well as available *in vitro* permeability models and PBPK models require more validation.

Finally, in certain areas, the appropriate technologies are not available or are in very early stages of development, where their suitability or applicability cannot yet be evaluated. In these areas of so-called complete gaps (category 3, Table 7) particular effort of scientific community including academic institutions could help to develop technological solutions. In the area of biological characterisation, such critical gaps include sophisticated *in vitro* models that could better mimic complex interactions of the immune system components and alternatives for LAL-based assays for evaluation of endotoxin contamination. Regarding the PCC characterisation analytical solutions are not available for the evaluation of surface properties of the pristine nanomaterials (surface area in liquids, surface coating quantification) and of the surface properties resulting from the NP interactions with biological media (protein corona formation). Recently, the establishment of new formulations, *e.g.*, lipid nanoparticles (LNPs) for delivery of complex biological drugs such as nucleic acids, is bringing to light additional needs in terms of analytical solutions for testing the integrity, payload, drug loading and release of nucleic acids and other biological complex drugs in delivery systems. Although an exhaustive comparison of all available assays is beyond the scope of this work, in Table 8 below we highlighted some specific sub-challenges within the five categories of characterisation listed previously, exemplified by a comparison of liposomal doxorubicin as a ‘classical’ nanomedicine *versus* an mRNA-based, LNP-formulated therapeutic as a novel class. It should be noted that although both these formulation types are based on non-covalent assembly of lipids, and both typically incorporate cholesterol, physiological phospholipids and a PEGylated lipid, they still require very different analytical approaches, largely because of their fundamentally different APIs. We would like to emphasize the need for method development and standardisation within the field of nucleic acid-based therapeutics, as these are very often nanotechnology-based, and currently a field of intense research in the wake of the successful Covid-19 vaccines.

**Table 8:** Examples of formulation-specific methodological gaps in two types of lipid-based nanomedicinal products.

Category of characterisation	Liposomal doxorubicin	mRNA/LNP therapeutic
Surface properties	*Gap:* PEGylation density and homogeneity.	*Gap:* PEGylation density and homogeneity; degree of PEG shedding over time.
Drug loading & release	HPLC-UV, LC-MS/MS yields good sensitivity and specificity.	*Gap:* Simultaneous quantification and verification of integrity of mRNA is challenging; fluorescence assays prone to interference, amplification-based assays prone to bias. Free mRNA cannot be separated by ultrafiltration.
Kinetic properties in biological media (drug release)	Ultrafiltration and stable isotope labelling with LC-MS/MS	*Gap:* Extraction and detection of mRNA (quantification, verification of integrity) in presence of endogenous biomolecules is very challenging.
ADME & biodistribution	Drug and metabolites are easily detectable in blood, tissue and excretions with good sensitivity by LC-MS/MS	*Gap:* Detection of mRNA (quantification, verification of integrity) in presence of endogenous RNA and other biomolecules is highly challenging. Instability of RNA adds to the challenge. *In silico* modelling of pharmaceutical effect needs to account for translation of the API.
Interaction with blood & immune systems	*Gap:* Doxorubicin absorbance interference in chromogenic assays for interaction with the immune system.	*Gap:* Immune cell activation can be highly dependent on mRNA sequence and presence of minor immunogenic contaminants, *e.g.* dsRNA or DNS/RNA hybrids.

## 6 Conclusions

Mutual acceptance of data by regulatory agencies is key for making innovative nanotechnology-based products available worldwide. Furthermore, the definition of standards that are accepted by the various regulatory bodies would help to reduce the uncertainty for product developers and ensure market authorisation for this emerging product class in different geographical regions.

Within this study, we have systematically analysed and compiled the information needs currently published by the regulatory authorities in Europe, US and Japan. Now it is of utmost importance that the regulatory and scientific communities agree on methods that can provide the required data and define common standardisation needs. In order to support this prioritisation process, we have compiled currently available methods and categorised them according to their level of maturity. Furthermore, we have also highlighted areas where methodological gaps exist and focussed research activities should be initiated by research institutions supported by test development programmes.

However, the challenges often lie in the details. The characterisation of physicochemical properties of nanotechnology-based health products requires very specific methods that need to be optimized/adapted to each nanotechnological platform, raising the question on the level of specificity/flexibility that can be accepted or is required for a standardised method, without the necessity to develop multiple standards in the same area. Another challenge is related to the standardisation of biological tests *e.g.* immunological assays, which are characterised by a high variability of results reflecting inter-individual variations of immune responses. Such variability can make corresponding methods quite difficult to validate and standardise. Finally, lack of reliable humanised models for the prediction of biological effects is a limiting factor for the proper investigation of pharmacokinetic profile of nanotechnology-based products, which can be significantly different from small-molecule products.

A question also arises on which of the standardisation pathways is the most relevant for nanotechnology-based health products. Whereas ISO standards are applicable to medical devices, the Ph. Eur. is a reference for the quality assessment of medicinal products in Europe. Many *in vitro* toxicity assays are being standardised under OECD, but they do not apply to medicinal products. A number of physicochemical methods and *in vitro* immunological assays addressing nanomedical products have been taken on board by ASTM International, but for the moment their formal recognition by European authorities is lacking.

Considering the complex and different regional procedures leading to the regulatory acceptance of methods, a concerted action by all parties for the prioritisation of methods and agreement on standardisation requirements is needed. The regular meetings of the Global Summit on Regulatory Science (GSRS) offer a platform for discussion and exchange for decision makers, regulatory scientists and standardisation bodies [76]. More such effort should be undertaken to develop a common standardisation pathway that could be relevant for nanotechnology-based products in different sectors and different geographical regions making the standardisation activities more efficacious and harmonised.

## Disclaimer

The views and opinions expressed in this report are those of the authors and do not necessarily reflect the official position of their organisations.

## Appendix A Supplementary data

Supplementary material Image 1

## Footnotes

1 NCI-NCL SOPs available at: https://ncl.cancer.gov/resources/assay-cascade-protocols

2 EUNCL SOPs available at: www.euncl.eu
