# Supplementary material for: Methodological needs in the quality and safety characterisation of nanotechnology-based health products: Priorities for method development and standardisation
Source: J Control Release. 2021 Aug 10;336:192–206. doi: 10.1016/j.jconrel.2021.06.016 (PMC8390938; doi:10.1016/j.jconrel.2021.06.016)
Supplement: Supplementary file 1 — Supplementary material [file mmc1.docx]

**Supplementary Material**

**Table S1**: Regulatory documents specifically addressing nanotechnology-based health products and corresponding product categories; EMA: European Medicines Agency, FDA: Food and Drug Administration (USA), MHLW: Ministry of Health, Labour and Welfare (Japan), SCENIHR: Scientific Committee on Emerging and Newly Identified Health Risks, ISO: International Standardisation Organisation.

| Category of product | Examples of products | Documents addressing nanotechnology- based medical products |
| --- | --- | --- |
| All products including health care products | | |
| Consumer products including medical products | Electronics, cosmetics, textiles, health products | - SCENIHR. Risk assessment of products of nanotechnologies; 2009 |
| Products containing nano-silver | Wound dressing with silver nanoparticles | - SCENIHR. Nanosilver: safety, health and environmental effects and role in antimicrobial resistance; 2014 |
| Medicinal products | | |
| Nanotechnology-based medicinal products | Albumin-bound paclitaxel NP, Glatiramer acetate | - EMA Reflection paper on nanotechnology-based medicinal products for Human Use. EMEA/CHMP/79769/2006 - FDA/CDER. Guidance for Industry: Drug Products, including Biological Products, that Contain Nanomaterials: (2017) (draft guidance) |
| Liposomal products | Liposomal doxorubicin,  Liposomal daunorubicin,  Pegylated liposomal irinotecan,  Liposomal Amphotericin B,  Liposomal Hepatitis A vaccine | - EMA/CHMP. Reflection paper on the data requirements for intravenous liposomal products developed with reference to an innovator liposomal product. London; 2013; EMA/CHMP/806058/2009/Rev.02. - FDA/CDER. Guidance for Industry. Liposome Drug Products: Chemistry, Manufacturing, and Controls; Human Pharmacokinetics and Bioavailability; and Labeling Documentation (2018) - MHLW. Guideline for the development of liposome drug products (2016) |
| Block copolymer micelle products | Peginterferon alpha-2a,  Pegaspargase  Sevelamer | - EMA/CHMP/ MHLW. Joint MHLW/EMA reflection paper on the development of block copolymer micelle medicinal products. London; 2013; EMA/CHMP/13099/2013. |
| Iron based nano-colloidal products | Ferumoxytol,  Ferric carboxymaltose | - EMA/CHMP. Reflection paper on the data requirements for intravenous iron-based nano-colloidal products developed with reference to an innovator medicinal product. London; 2015; EMA/CHMP/SWP/620008/2012. |
| Nucleic acid-loaded nanotechnology-based drug products | siRNA-loaded and mRNA-loaded lipid and polymeric NPs | - MHLW. Reflection paper on nucleic acids (siRNA)-loaded nanotechnology based drug products (2016) |
| Coated nanomedicine products | PEGylated liposomes, products with targeting ligand on the surface | - EMA/CHMP. Reflection paper on surface coatings : general issues for consideration regarding parenteral administration of coated nanomedicine products. London; 2013; EMA/325027/2013. |
| Medical devices | | |
| Medical devices containing nanomaterial | Nanostructured hydroxyapatite  Nano-ceramic dental composite, tumour tracers | - SCENIHR. Opinion on the guidance on the Determination of Potential Health Effects of Nanomaterials Used in Medical Devices; 2015 - ISO TR 10993-22: Biological Evaluation of medical devices – Part 22: Guidance on nanomaterials. |

**Tables S2-S4 Compilation of methods addressing regulatory information needs for nanotechnology-enabled health products.**

**Compatibility score:** 1= very specific method only applicable to one specific NPl/API combination or that has not been tested yet on other nanomaterials; 2= method applicable to a specific nanomaterial type or API class (e.g. AuNPs, mRNAs, liposomes); 3= method applicable to multiple nanomaterial types (e.g. inorganic or organic carriers) with some specific limitations related to their physicochemical properties (e.g. size, solubility, optical properties); 4= method applicable to all types of nanomaterials with some specific limitation related to their physical-chemical properties (e.g. positive surface charge), 5= method applicable to all the NP/API classes.

**Status of validation score**: 1 = method exists as primary publication or proof-of-concept; 2= Method in regular use in one lab for research purposes but not officially validated; 3= Method used routinely in relevant R&D environment, if intra-laboratory variability was assessed in at least one laboratory; 4= Method fully qualified (inter-laboratory variability assessed) but no formal standard exists; 5=standardised method (ISO, CEN, ASTM, OECD).

**Table S2:** Compilation of methods addressing regulatory information needs for nanotechnology-enabled health products related to physicochemical properties and stability in biological complex media. **Abbreviations:** HPLC-CAD = High-Performance Liquid Chromatography- Charged Aerosol Detector, CAD= Charged Aerosol Detector, ELSD= Evaporative Light Scattering Detector , MS= Mass Spectrometry, TGA= Thermogravimetric Analysis, FTIR= Fourier-Transform Infrared Spectroscopy, BET= Brunauer-Emmett-Teller , NMR= Nuclear Magnetic Resonance , LC-MS/MS= Liquid Chromatography with Tandem Mass Spectrometry, AF4-UV-VIS-MALS-DLS= Asymmetric-Flow Field Flow Fractionation coupled with UV-Visible, Multiangle Light Scattering and Dynamic Light Scattering detectors online, ICP-MS= Inductively Coupled Plasma Mass Spectrometry.

| **Category** | **Subcategory** | **Endpoint/measurand** | **Method Description (readout)** | **Reference** | **Compatibility score** | **Status of validation** | **Applicability/limitations** |  |
| --- | --- | --- | --- | --- | --- | --- | --- | --- |
| Surface properties | Coating analysis | Total PEG concentration (w/v), as well as the bound and free unbound fractions (w/v) | Centrifugation/HPLC-CAD or HPLC-ELSD | NCI-NCL (PCC16) | 1 | 3 | Applicable to gold nanoparticles only |  |
|  |  | Total amount of PEG/surface coating | HPLC-CAD/ELSD/MS | EUNCL (PCC33) | 1 | 2 | Applicability to gold nanoparticles only |  |
|  |  | Amount of coating on the NP surface | TGA | NCI-NCL(PCC-17) | 3 | 3 | Applicable to metal and ceramics. High limit of detection, may not allow identification of chemical identity of the coating |  |
|  |  | Qualitative evaluation of functionalities on the NP surface | FTIR | ISO/TS 14101:2012 | 1 | 5 | Applicability to gold nanoparticles |  |
|  |  | Homogeneity of functionality/ surface distribution of ligands | No technological solutions available | | | | | |
|  | Surface hydrophobicity | Relative hydrophobicity | Binding affinity to a collector | under standardisation by OECD | 2 | 3 | Applicable to noble metal particles (> 10 nm) and liposomes (> 70 nm) |  |
|  | Surface area | SSA | BET | [ISO 9277:2010](https://www.nanoobjects.info/de/projekte/era-net-siinn/nanoximet) | 3 | 5 | Only for inorganic NP powders |  |
|  |  |  | NMR | NanoReg Toolbox | 3 | 2 | Applicable to soft nanoparticles. Validation status is unknown |  |
| Drug loading and release  (for delivery systems only) | Total drug loading | API total concentration (w/v) | LC-MS/MS | EUNCL (PCC-30) | 3 | 4 | Need for adaptation to each nanotechnological platform |  |
|  | Free drug fraction | Free *vs* encapsulated API concentration (w/v) | Ultrafiltration + HPLC, LC-MS/MS | EUNCL (PCC-31) | 3 | 4 |  |  |
|  |  |  | RP-HPLC‒ICP-MS | NCI-NCL (PCC-14) | 1 | 3 | Applicable to gadolinium-based contrast agents |  |
| Kinetic properties in complex biological media | Drug release in plasma | Drug released by the NP (w/v) vs time | Ultrafiltration + HPLC, LC-MS/MS. | NCI-CL (PHA-1&2) | 3 | 3 | Applicable to nanocarriers that can be separated from the API by ultrafiltration, and to APIs that can be detected by mass spectrometry |  |
|  | Physical stability in biological media | NP size changes vs time | AF4-UV-VIS-MALS-DLS | EUNCL (PCC-22) | 4 | 4 | Need for adaptation to each nanotechnological platform. |  |
|  |  |  | Batch DLS | EUNCL (PCC-21) | 4 | 4 |  |  |
|  |  |  | Analytical ultracentrifugation | EUNCL (PCC-24) | 4 | 2 |  |  |
|  |  |  | Particle tracking analysis | EUNCL (PCC-23) | 4 | 2 |  |  |
|  | Protein corona formation | Amount of proteins on the particle surface (qualitative) and/or corona composition | Plasma preparation, SDS-PAGE, LC-MS/MS analysis of the SDS gel for composition [R2] | NCI-NCL (ITA-4) | 3 | 4 | Limited to particles that can be separated from plasma by centrifugation |  |
|  |  |  | LC-MS/MS or LC-UV-VIS | Scientific literature [1–3] | 4 | 2 | Inorganic and large (or dense) organic particles. |  |
|  | Chemical stability *vs* time | Degradation product(s) *vs* time. Qualitative and quantitative assessment. | Chromatographic methods | ICH-Q1 | 1 | Depending of the NP/API | Highly specific for each combination of NP/API |  |

**Table S3**: Compilation of methods, *in vitro* and *in silico* models addressing regulatory information needs for nanotechnology-based health products related to ADME and biodistribution.

| **Subcategories** | **Endpoint** | **Available techniques/models** | **Used for nanomaterials?** | **Status of validation** | **Limitations** |
| --- | --- | --- | --- | --- | --- |
| Detection and quantification in biological matrices | Quantification of API | LC-MS/MS, ICP-MS, UV-spectroscopy | Yes | 3 or 4 | Requires prior extraction of API from delivery system |
|  | Quantification of nanomaterial | Imaging (for labelled NPs),  ICP-MS | Certain types of nanomaterials only | 1 or 2 | Imaging requires adequate particle labelling, ICP-MS is specific only for certain types of nanomaterial |
| *In vitro* models | Protein aggregation | Cell free assays (equilibrium dialysis, fractionation-based assays) | Yes | 1 or 2 |  |
|  | Cell uptake, Gut absorption, paracellular and transcellular transport pathways, metabolism | Caco-2 |  |  | Cancer cells physiology and reaction may significantly differ from normal human cells |
|  | Renal absorption, metabolism, transport across tight junction-barrier | MDCK |  |  |  |
|  | Vascular permeability | HUVEC (primary cells), EaHy926 (immortalised cell line) |  |  | Use of primary cells is associated with high variability of responses |
|  | Metabolism and elimination | Primary hepatocytes |  |  |  |
| *In vivo* models | Tissue distribution – API concentration or NP number per organ (by volume or weight) | Mouse and rat models used extensively | Yes | 3 | Not optimal to assess PK profiles in tumour |
| *In silico* models | Computational prediction of biodistribution | Existing models for blood, liver, lungs, spleen, kidney, brain, gut, heart | Yes | 2 | Need to be optimised to each platform individually. |

**Table S4**: Compilation of available *in vitro* methods addressing regulatory information needs for nanotechnology-based products related to the interaction with blood and immune system.

| **Subcategories** | **Endpoint/Measurand** | **Name of the method** | **Biological model** | **Reference** | **Designed for nanomaterials?** | **Status of validation** | **Specific limitations** |
| --- | --- | --- | --- | --- | --- | --- | --- |
| Endotoxin contamination | Endotoxin quantification | Limulus amebocyte lysate (LAL) test | NA | Ph. Eur. 2.6.14 | NO | 5 | Subject to interference with nanoparticles |
|  |  | Test for bacterial endotoxins using recombinant factor C | NA | Ph. Eur. 2.6.32 | NO | 5 | Applicability to nanomaterials unknown |
|  |  | Limulus amebocyte lysate (LAL) test | NA | EN ISO 29701:2010 | Yes | 5 | Subject to interference with nanoparticles |
|  |  | Test for bacterial endotoxins using LC-MS on fatty acids | NA | H2020 REFINE | Yes | 1 |  |
|  | Pyrogen detection | Monocyte Activation Test (MAT) (also inflammation) | Monocytes/whole blood | Ph. Eur. 2.6.30 | NO | 5 | Not suitable for cytotoxic compounds |
| Hemocompatibility | Release of haemoglobin (Haemolysis) | Standard test method for analysis of haemolytic properties of nanoparticles | Whole blood | ASTM E2524 - 08(2013) | Yes | 5 |  |
|  | Platelet aggregation | Platelet aggregation by cell counting | Plasma platelets | NCI-NCL /EUNCL ITA-2.1 | Yes | 3 / 4 |  |
|  |  | Platelet aggregation by light transmission | Plasma platelets | NCI-NCL/EUNCL ITA-2.2 | Yes | 3/ 4 |  |
|  | Plasma coagulation (prothrombin time, activated partial thromboplastin time and thrombin time) | Analysis of nanoparticle effects on plasma coagulation times in vitro | Plasma | NCI-NCL/EUNCL ITA-12 | Yes | 3 / 4 |  |
|  | Platelet adhesion and fibrin polymerisation | Activation of the clotting system | Heparinised human plasma. | Scientific publication [4] | Yes | 1 /2 | Insufficient maturity |
| CARPA and Complement activation | Quantification of iC3b  (all pathways) | New test method for assessing the activation of the complement system through quantification of iC3b concentration by ELISA | Human plasma | ASTM WK69051, NCI-NCL/EUNCL  ITA-5.2 | Yes | 3 |  |
|  | Basophil/mast cell activation | Evaluation of basophilic and mast cell markers of activation | Basophilic leukaemia cells (KU812) | H2020 REFINE | Yes | 2 | Insufficient maturity |
| Effect on inflammation and innate immune cells | NP uptake by phagocytes | Evaluation of nanoparticulate material internalization by phagocytic cells in vitro | Promyelocytic leukaemia cells (HL-60) | ASTM WK60553, NCI-NCL  ITA-9.-1 | Yes | 4 | interference with luminol-dependent chemiluminescence. |
|  | Effect on macrophage function | M1/M2 Assay: Evaluation of the effect on pro-inflammatory (M1) vs. anti-inflammatory/pro-fibrotic (M2) macrophages | Monocytes/whole blood | H2020 REFINE | Yes | 1 | Insufficient maturity |
|  | Phagocytosis of apoptotic target cells | Phagocytosis assay with M-CSF activated HMDM and TAMRA-labelled  target cells | Primary human macrophages | H2020 NANOMMUNE 3.02 | Yes | 2 | Risk of Interference with fluorescence measurement, validation status unknown |
|  | Production of Reactive Oxygen Species (ROS) | 5-(and 6)-Chloromethyl-2’,7’ Dichloro-dihydrofluorescein diacetate (CM-H2DCF-DA) assay for evaluating NP-induced intracellular ROS production | Murine macrophage cell line (RAW264.7) | ISO/TS 19006:2016 | Yes | 5 | Risk of Interference with fluorescence measurement, non-human cell line |
|  | Chemoattractant capacity | Quantitative measurement of the chemoattractant capacity of a nanoparticulate material in vitro | Promyelocytic leukaemia cell line (HL60) | ASTM WK60373  NCI-NCL  ITA-8.-1 | Yes | 3 or 4 |  |
|  | Inflammasome activation | Evaluation of effect on pro-inflammatory cytokines IL-1beta and IL-18 | Human monocytes cell line (THP1) | H2020 REFINE | Yes | 2 |  |
|  | Natural Killer cell activity (Lysis of tumorous target cells) | Measurement of nanoparticle effects on cytotoxic activity of NK cells by label-free RT-CES system | Cell line (NK92) | NCI-NCL  ITA-11 | Yes | 3 or 4 | Requires a dedicated instrument for the real time cell electronic system |
| Effect on adaptive immune system | Dendritic Cell (DC) maturation | Effect on DC maturation | Peripheral blood monocytes | H2020 REFINE | Yes | 2 |  |
|  |  | Analysis of Nanoparticle Effects on Maturation of Monocyte Derived Dendritic Cells In Vitro | Peripheral blood monocytes | NCI-NCL  ITA-14 | Yes | 3 |  |
|  | Effect on lymphocytes | Human leukocyte proliferation assay (HuLa) | PBMC (human blood) | NCI-NCL (ITA-18) /EUNCL | Yes | 3 |  |
|  | Antibodies production | Detection of naturally occurring antibodies to PEG and PEGylated liposomes | Plasma | NCI-NCL | Yes | 3 |  |

References:

[1] M.P. Monopoli, A.S. Pitek, I. Lynch, K.A. Dawson, Formation and characterization of the nanoparticle-protein corona, Methods Mol. Biol. 1025 (2013) 137–155. https://doi.org/10.1007/978-1-62703-462-3_11.

[2] D. Docter, U. Distler, W. Storck, J. Kuharev, D. Wünsch, A. Hahlbrock, S.K. Knauer, S. Tenzer, R.H. Stauber, Quantitative profiling of the protein coronas that form around nanoparticles, Nat. Protoc. 9 (2014) 2030–2044. https://doi.org/10.1038/nprot.2014.139.

[3] A.J. Chetwynd, K.E. Wheeler, I. Lynch, Best practice in reporting corona studies: Minimum information about Nanomaterial Biocorona Experiments (MINBE), Nano Today. 28 (2019) 100758. https://doi.org/10.1016/j.nantod.2019.06.004.

[4] J. Sanchez, G. Elgue, R. Larsson, B. Nilsson, P. Olsson, Surface-adsorbed fibrinogen and fibrin may activate the contact activation system, Thromb. Res. 122 (2008) 257–263. https://doi.org/10.1016/j.thromres.2007.11.008.
